# Synthesis and Cytotoxicity of Novel Hexahydrothieno-cycloheptapyridazinone Derivatives

**DOI:** 10.3390/molecules14093494

**Published:** 2009-09-09

**Authors:** Amedeo Pau, Gabriele Murineddu, Battistina Asproni, Caterina Murruzzu, Giuseppe E. Grella, Gérard A. Pinna, Maria M. Curzu, Irene Marchesi, Luigi Bagella

**Affiliations:** 1 Dipartimento Farmaco Chimico Tossicologico,Università di Sassari, Via Muroni 23/A, 07100 Sassari, Italy; 2 Department of Biomedical Sciences, Division of Biochemistry and Biophysics, and National Institute of Biostructures and Biosystems, University of Sassari, Viale San Pietro 43/b 07100 Sassari, Italy

**Keywords:** pyridazinones, synthesis, cytotoxicity

## Abstract

Designed as a new group of tricyclic molecules containing the thienocycloheptapyridazinone ring system, a number of 2*N*-substituted-hexahydrothieno-cycloheptapyridazinone derivatives were synthesized and their biological activity evaluated. Among the synthesized compounds, derivatives **7d** and **7h** were found to possess cytotoxic activity against non-small cell lung cancer and central nervous system cancer cell lines, respectively.

## 1. Introduction

Among diseases, cancer is not a single pathological state but a broad group of diseases characterized by a high proliferative index and the spread of aberrant cells from their site of origin [1]. Clinically, the therapeutic treatment of cancer is a combination of surgery and/or radiotherapy with chemotherapy [2,3].

Current chemotherapy consists of cytotoxic (cell-killing) agents and anti-hormonal drugs, which reduce the proliferation of the tumors [2,3]. The therapeutic use of anticancer drugs is complicated by systemic toxicity, usually observed in the bone narrow, the gastrointestinal (GI) tract and hair, and by development of resistance. Therefore, the search for novel chemical structures with broader therapeutic windows and acceptable resistance profiles is being actively pursued.

In discovering anticancer compounds, a notable role is played by polycondensed heterocycles containing the pyridazinone moiety [4]. A wide spectrum of pharmacological activities has been reported for these compounds. These include anticancer [5], antihypertensive, anti-thrombotic and antiulcerative properties [6,7,8,9]. Pyridazinone derivatives also possess affinity for benzodiazepine receptors [10] and the ability to inhibit the human matrix metalloproteinase [11] and aldose reductase [12,13] enzymes.

**Figure 1:**
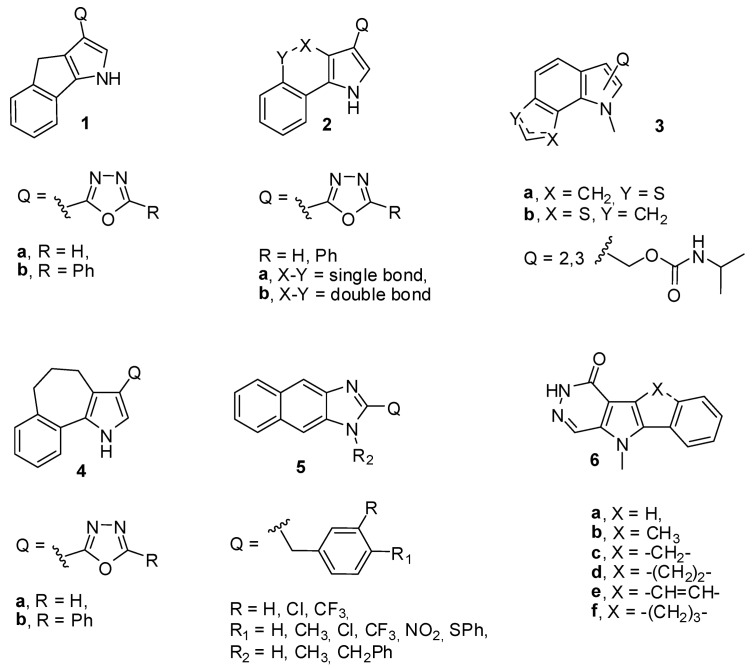
Chemical structures of some known anticancer agents synthesized by our group.

A major interest in our group is the design, synthesis and evaluation of new antiproliferative compounds as candidate cytotoxic and anticancer agents. In recent years, we have reported the synthesis of novel derivatives of 1,4-dihydroindeno[1,2-*b*]pyrroles (**1**) [5], 1*H*-benzo[*g*]indoles (**2**) [5], thieno[3,2-*g*]indoles (**3**) [14], 1,4,5,6-teterahydrobenzo[6,7]cyclohepta[1,2-*b*]pyrroles (**4**) [5], naphto[2,3-*d*]imidazoles (**5**) [15], pyrrole[2,3-*d*]pyridazinones (**6**) [16] and their cytotoxic activities in the NCI preclinical antitumor screen (Figure 1).

In continuation of our research in this field, we describe herein the synthesis of novel 2,4,4a,5,6,7-hexahydro-3*H*-thieno[2’,3’:6,7]cyclohepta[1,2-*c*]pyridazinones bearing substituted piperazine, piperidine and morpholine moieties, using a random screening approach [17], and the antitumor activities of the resulting compounds **7a-l **reported in Table 1.

**Table 1:** Novel thienocyclohepta[1,2-*c*]pyridazinones **7a-l**. 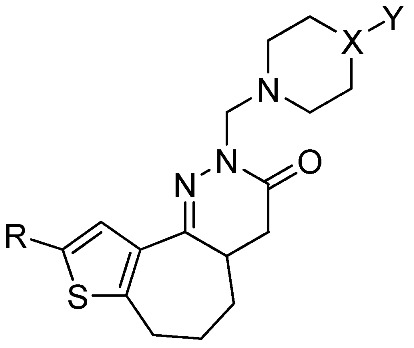

7	a	b	c	d	e	f	g	h	i	j	k	l
R	H	H	H	H	CH_3_	CH_3_	CH_3_	CH_3_	H	CH_3_	H	CH_3_
X	N	N	N	N	N	N	N	N	CH_2_	CH_2_	O	O
Y	Ph	*o*-OCH_3_-Ph	*o*-F-Ph	CH_3_	Ph	*o*-OCH_3_-Ph	*o*-F-Ph	CH_3_	CH_3_	CH_3_	-	-

## 2. Chemistry

**Figure 2:**
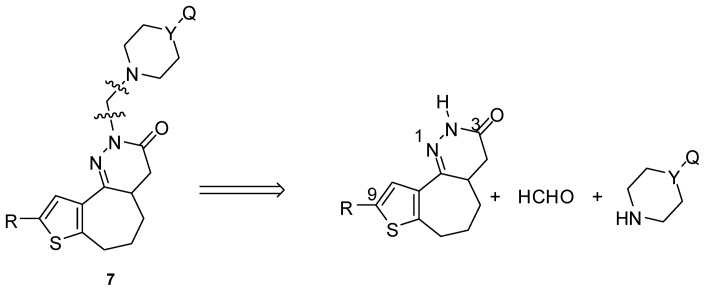
The retrosynthetic analysis of the target compounds **7**.

The retrosynthetic analysis shown in Figure 2 shows how novel pyridazinone derivatives could be prepared by condensation of a tricyclic ring system with formaldehyde and the appropriate substituted piperazine synthon or its isosteres such as methylpiperidine and morpholine.

**Scheme 1:**
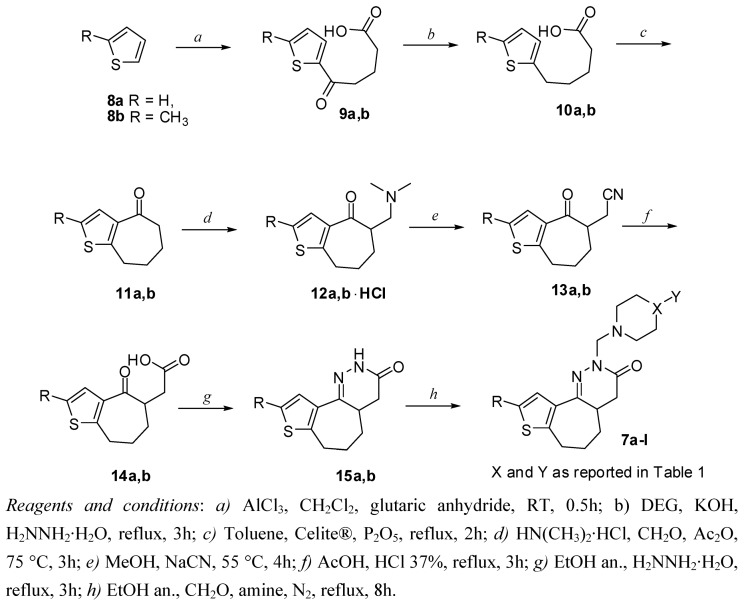
Synthesis of novel pyridazinone derivatives **7a-l**.

Accordingly, the new 2,4,4a,5,6,7-exahydro-3*H*-thieno[2’,3’:6,7]cyclohepta[1,2-*c*]pyridazin-3-one derivatives **7a-l** were synthesized in according to Scheme 1. The reaction of thiophenes **8a,b** with glutaric anhydride to give ketoacids **9a,b **was followed by Wolff- Kishner reduction to **10a,b**, whose cyclization with P_2_O_5_ over Celite® gave the ketones **11a,b**. A Mannich reaction furnished **12a,b**, which were converted with NaCN in CH_3_OH into the nitriles **13a,b**. Hydrolysis of these nitrilesin refluxing HCl/AcOH led to the γ-ketoacids **14a,b**; condensation of the latter with hydrazine hydrate afforded the pyridazinones **15a,b**.

Preparation of the target compounds **7a-l **was accomplished by treatment of the pyridazinones **15a,b **with formaldehyde and appropriate amines.

## 3. Results and Discussion

A new series of twelve substituted pyridazinones **7a-l** were synthesized and eight of them (**7c-e,h-l**)were evaluated at a single concentration of 10^-5^ M (10 μM) for their antitumor activities. The evaluation established a primary screening where compounds were tested to determine their growth inhibitory properties against sixty different human tumor cell lines *in vitro* [18,19,20]*.* The compounds were added at a single concentration and the cell culture was incubated for 48 h. End point determinations were made using a protein binding dye, sulforhodamine B (SRB), which was used to estimate cell viability or growth [21]. The results for each compound are reported as percent growth of treated cells when compared to untreated control cells (Table 2, Table 3). Range of growth % shows the lowest and the highest growth % found among different cancer cell lines, where all tested compounds have demonstrated being scarcely active or completely inactive in the antitumor screening *in vitro* (Table 2). 5-Fluorouracil (5-FU) was used as reference compound with the mean growth inhibitory effect (GI_50_) of 2.45 × 10^-5^ M which corresponds in logarithmic scale to 4.61 [22]. 

**Table 2:** Anticancer screening data of selected pyridazinone derivatives **7c,d,e,h,i,j,k,l**.^a^

Panel/Cell Lines	Compounds							
Non-small cell lung cancer	7c	7d	7e	7h	7i	7j	7k	7l
Mean growth %	97.85	**98.09**	103.17	101.55	101.33	101.88	96.66	102.97
Range of growth %	81.03 to 113.17	72.06 to 120.26	78.91 to 108.81	92.87 to 109.80	80.20 to110.82	81.70 to 119.13	75.17 to 113.32	93.09 to 112.16
**Colon cancer**								
Mean growth %	107.29	105.03	109.01	104.88	103.17	104.91	101.79	123.28
Range of growth %	100.56 to 117.52	96.19 to 111.60	97.01 to 119.39	93.70 to 123.28	93.76 to 117.4	95.50 to 112.76	93.53 to 114.38	99.22 to 110.16
**Breast Cancer**								
Mean growth %	103.25	100.59	107.91	105.20	104.68	107.49	101.71	108.39
Range of growth %	92.60 to 113.98	91.34 to 109.30	96.71 to 121.53	91.23 to 120.16	88.76 to122.29	91.22 to 124.23	94.33 to 110.47	87.28 to 129.10
**Ovarian Cancer**								
Mean growth %	104.72	101.64	108.09	102.84	109.28	102.64	103.69	104.24
Range of growth %	99.67 to 111.88	98.15 to 108.92	95.14 to 119.38	96.73 to 120.49	92.31to 170.96	94.48 to 117.11	92.28 to 117.79	96.32 to 122.92
**Leukemia**								
Mean growth %	100.46	**92.31**	98.55	102.95	94.13	100.01	86.67	98.54
Range of growth %	91.68 to 107.61	81.94 to 113.17	91.36 to 111.53	85.24 to 120.85	80.99 to	90.34 to 112.16	76.07 to 94.26	75.05 to 112.55
**Renal Cancer**								
Mean growth %	101.77	101.13	103.67	**95.02**	103.58	100.19	104.36	101.80
Range of growth %	93.09 to 110.89	95.38 to 108.40	94.16 to 114.16	77.38 to 114.45	94.14 to113.75	86.51 to 121.05	93.91 to 114.99	85.76 to 112.31
**Melanoma**								
Mean growth %	106.50	102.46	106.62	101.06	102.95	107.10	101.57	104.80
Range of growth %	100.28 to110.39	88.88 to 111.60	101.92 to116.24	92.73 to 112.29	87.54 to119.80	92.35 to 124.26	91.52 to 114.53	93.82 to 112.20
**Prostate Cancer**								
Mean growth %	102.25	100.46	114.38	109.08	101.76	99.92	103.85	109.31
Range of growth %	98.48 to 106.02	93.16 to 107.76	108.08 to 120.68	104.25 to 113.91	99.78 to 103.76	96.26 to 103.58	102.00 to105.70	102.40 to 116.22
**CNS Cancer**								
Mean growth	99.49	100.85	100.78	**82.03**	111.34	98.38	107.23	97.20
Range of growth %	91.08 to 108.91	77.31 to 131.16	89.96 to 110.76	72.14 to 115.77	80.17 to 194.64	85.30 to 105.97	81.54 to 164.17	82.07 to 106.60

^a^ Assay at 1-dose 10^-5 M^ (10 µM) concentration.

Nevertheless, compounds **7d** and **7h **displayed a higher anti-proliferative activity in the non-small cell lung cancer cell line EKVX and in the CNS cancer cell line SNB-75, which showed growth inhibitions of 27.94% and 27.86%, respectively (Table 3). Compounds **7k** and **7l **also showed cell growth inhibitory activity, even if weaker than the one expressed by **7d** and **7h**. In particular, compound **7l** was found to be active as growth % inhibitor of the leukemia cell line RPMI-8226 with a value of 24.95%; the derivative **7k** was active on leukemia cell line SR with a value of 24.83% and non-small cell lung cancer cell line EKVX with a value of 23.93%. Moreover, **7e** was found to be active as growth % inhibitor of the non-small cell lung cancer cell line EKVX with a value of 21.09%. Finally, **7c** and **7i** showed a growth % inhibitor of non-small cell lung cancer cell line HOP-92 with values of 18.97, and 19.80%, respectively.

**Table 3:** *In vitro* cancer lines growth % inhibition of pyridazinones **7d,e,h,k,l**.

Compd			Panel/Cell Lines					
	Non-small cell lung cancer			Leukemia		Renal cancer		CNS cancer
	EKVX*	HOP-92		SR	RPMI-8226	CAKI-1	UO-31	SNB-75*
**7d **	27.94	20.09						22.69
**7e **	21.09							
**7h **						22.62	22.23	27.86
**7k**	23.93			24.83				
**7l **					24.95			

* The most sensitive cell lines.

## 4. Conclusions

As part of our continuous search for potential biologically active compounds, a series of pyridazinone derivatives were synthesized and assessed for their anticancer activity. It was found that all new eight compounds tested showed weak or incomplete activity without significant differences between 9-substituted and unsubstituted derivatives. Specifically, two of them showed scant activity, while others showed no activity in the cell growth inhibition assay against sixty different human cancer cell lines panel *in vitro*. From these data, we may conclude compounds **7d **and **7h **were the most effective molecules for anti-proliferative activity, specifically in non-small cell lung cancer and CNS cancer respectively, so they might be useful as leads for designing new compounds with potential antitumoral activity. This structure was derived from pyridazinone with hydrogen or methyl group in the 9-position linked to the 4-methylpiperazine moiety by a methylene spacer. The obtained results prove the necessity for further investigations to clarify the molecular mechanisms involved in antitumor activities to acquire more information about the structural requirements for enhancing anticancer activities and minimizing neurotoxicities, the synthesis of more new derivatives with different substituents at other positions is needed.

## 5. Experimental

### 5.1. General

Melting points were determined using a Reichert-Köfler hot-stage apparatus and are uncorrected. Infrared spectra were recorded with a Perkin-Elmer Paragon 500 FT IR spectrophotometer (KBr pellets, in Nujol mulls, as well as in film). ^1^H-NMR spectra were recorded on a Varian XL 200 FT NMR spectrometer using CDCl_3_ as solvent, unless otherwise specified. Chemical shifts are reported in δ or ppm and coupling constants (*J*) in Hertz (Hz), downfield from tetramethylsilane (TMS). Multiplicities are recorded as s (singlet), br s (broad singlet), d (doublet), t (triplet), q (quartet), m (multiplet). Reactions were monitored by analytical thin-layer chromatography (TLC) using SiO_2_ Polygram SIL and ALOX N/UV_254_ precoated plastic sheets and with visualization by irradiation with a UV lamp and/or iodine vapor for detection. Flash chromatography was performed using Merck silica gel type 60 (230-400 mesh ASTM). Electron ionization mass spectra (70 eV) were recorded on a Hewlett-Packard 5790-5970 MSD gas chromatograph/mass spectrometer. Atmospheric Pressure Ionization Electrospray (APIES) mass spectra, when reported, were obtained on a Agilent 1100 series LC/MSD spectrometer. All moisture sensitive reactions were performed under nitrogen atmosphere, using oven-dried glassware. Anhydrous DCM, THF and DMF was obtained from Aldrich, Lancaster or Merck. All starting materials and reagents were commercially available from Aldrich, Lancaster and Avocado. Evaporation was performed *in vacuo* (rotary evaporator). Anhydrous sodium or magnesium sulfate was always used as the drying agent. Elemental analyses were performed in a Perkin-Elmer 240C elemental analyzer, and the results were within ± 0.4% of the theoretical values, unless otherwise noted.

### 5.2. General procedure for the synthesis of 5-oxopentanoic acids **9a,b**

To a suspension of anhydrous AlCl_3_ (17.54 mmol) in dry CH_2_Cl_2_ (20 mL) cooled with an ice bath, a solution of glutaric anhydride (19 mmol) in dry CH_2_Cl_2_ (20 mL) was added dropwise under a N_2 _atmosphere, and the whole mixture was stirred at RT for 0.5 h. Then a solution of thiophene **8a,b** (17 mmol) in dry CH_2_Cl_2_ (15 mL) was added dropwise and the reaction mixture stirred at the same temperature for an additional 0.5 h. The mixture was poured into crushed ice and conc. HCl was slowly added followed by warming until the suspended materials dissolved. The aqueous phase was separated and extracted with CH_2_Cl_2_. The combined organic phase was washed with H_2_O and then extracted with 2N NaOH aqueous solution (5 × 7 mL): the solid separated upon acidification of the alkali layer, was filtered off and air dried to yield the desired product.

*5-(Thiophen-2-yl)-5-oxopentanoic acid* (**9a**): Yield 2.12 g (60%) as a cream solid: mp 93-94 °C; *R_f_*: 0.48 (CHCl_3_-MeOH 9:1); IR (Nujol, ν, cm^-1^): 1696 (COOH), 1654 (CO); ^1^H-NMR (CDCl_3_), *δ* ppm: 7.74 (d, 1H, *J =* 4 Hz, CH), 7.64 (d, 1H, *J =* 5.2 Hz, CH), 7.15 (t, 1H, *J =* 3.6 Hz, CH), 3.04 (t, 2H, *J =* 7 Hz, CH_2_), 2.53 (t, 2H, *J =* 7.4 Hz, CH_2_), 2.09 (quint., 2H, *J =* 7 Hz, CH_2_); GC-MS *m/z*: 198 (M^+^); Calcd for C_9_H_10_O_3_S: C, 54.53; H, 5.08; S, 16.17. Found: C, 54.42; H, 4.89; S, 16.08.

*5-(5-Methyl-2-thienyl)-5-oxopentanoic acid* (**9b**): Yield 2.07 g (60%) as a cream solid: mp 105-107 °C; *R_f_*: 0.65 (CHCl_3_-MeOH 9:1); IR (Nujol, ν, cm^-1^): 1693 (COOH), 1650 (CO); ^1^H-NMR (CDCl_3_), *δ* ppm: 7.54 (d, 1H, *J =* 3.6 Hz, CH), 6.80 (d, 1H, *J =* 3 Hz, CH), 2.95 (t, 2H, *J =* 7.4 Hz, CH_2_), 2.54 (s, 3H, CH_3_), 2.5 (t, 2H, CH_2_), 2.09 (qu, 2H, CH_2_); GC-MS *m/z*: 212 (M^+^); Calcd for C_10_H_12_O_3_S: C, 56.58; H, 5.70; S, 15.11. Found: C, 56.49; H, 5.63; S, 15.21. 

### 5.3. General procedure for the synthesis of pentanoic acids **10a,b**

A mixture of 5-oxopentanoic acid **9a,b** (2.00 g, 9.3 mmol), diethylene glycol (DEG, 24 mL), potassium hydroxide (0.035 mol) and hydrazine hydrate (0.045 mol) was refluxed with a Dean-Stark apparatus for 3 h. The solution, after cooling to RT, was poured into cold water (50 mL), washed with ether, acidified with 6 N HCl and then extracted with ether (4 × 5 mL). The organic layer was dried over anhydrous sodium sulfate, filtered and evaporated *in vacuo* to give the title compounds. 

*5-(2-thienyl)pentanoic acid* (**10a**): Yield 1.50 g (82%) as a yellow amber solid: mp 41 °C; *R_f_*: 0.72 (CHCl_3_-MeOH 9:1); IR (Nujol, ν, cm^-1^): 1702 (COOH); ^1^H-NMR (CDCl_3_), *δ* ppm: 7.11 (d, 1H, *J =* 4 Hz, CH), 6.93 (d, 1H, *J =* 4 Hz, CH), 6.79 (t, 1H, *J =* 3.8 Hz, CH), 2.77 (t, 2H, *J =* 6.6 Hz, CH_2_), 2.41 (s, 2H, *J =* 6.8 Hz, CH_2_), 1.74 (m, 4H, *J =* 3.6Hz, 2CH_2_); GC-MS *m/z*: 184 (M^+^); Calcd for C_10_H_12_O_3_S: C, 58.67; H, 6.56; S, 17.14. Found: C, 58.59; H, 6.49; S, 17.23.

*5-(5-Methyl-2-thienyl)pentanoic acid* (**10b**): Yield 1.50 g (82%) as yellow amber solid: mp 47-49 °C; *R_f_*: 0.72 (CHCl_3_-MeOH 9:1); IR (Nujol, ν, cm^-1^): 1693 (COOH); ^1^H-NMR (CDCl_3_), *δ* ppm: 6.54 (s, 2H, 2CH), 2.77 (t, 2H, CH_2_), 2.43 (s, 3H, CH_3_), 2.38 (m, 2H, CH_2_), 1.70 (m, 4H, 2CH_2_); GC-MS *m/z*: 198 (M^+^); Calcd for C_10_H_12_O_3_S: C, 60.57; H, 7.12; S, 16.17. Found: C, 60.49; H, 7.06; S, 16.12. 

### 5.4. General procedure for the synthesis of ketones **11a,b**

To a solution of pentanoic acid **10a,b** (13 mmol) in toluene (35 mL), were added Celite^®^ (4.52 g) and phosphorus pentoxide (23 mmol). The mixture was refluxed for 2 h, then cooled and filtered. The filtrate was washed with 5% aqueous NaHCO_3_ solution (2 × 5 mL), dried (Na_2_SO_4_), filtered and concentrated *in vacuo*, to give the ketones as oils.

*5,6,7,8-Tetrahydro-4H-cyclohepta[b]thiophen-4-one* (**11a**): Yield 0.78 g (59%) as a yellow brown oil; *R_f_*: 0.87 (CHCl_3_-MeOH 9:1); IR (film, ν, cm^-1^): 1665 (CO). ^1^H-NMR (CDCl_3_), *δ* ppm: 7.33 (d, 1H, *J =* 5.4 Hz, CH-2), 6.91 (d, 1H, *J =* 5.4 Hz, CH-3), 3.03 (t, 2H, *J =* 5.2 Hz, CH_2_-8), 2.65 (t, 2H, *J =* 6.8 Hz, CH_2_-5), 2.39 (s, 3H, CH_3_), 1.92-1.16 (m, 4H, 2CH_2_); GC-MS *m/z*: 166(M^+^); Calcd for C_9_H_10_OS: C, 65.02; H, 6.06; S, 19.29. Found: C, 65.08; H, 6.12; S, 19.21. 

*5,6,7,8-Tetrahydro-2-methyl-4H-cyclohepta[b]thiophen-4-one* (**11b**): Yield 0.79 g (59%) of the compound **7 **as yellow brown oil which was used for the next step without further purification. *R_f_*: 0.88 (CHCl_3_-MeOH 9:1); IR (film, ν, cm^-1^): 1663 (CO); ^1^H-NMR (CDCl_3_), *δ* ppm: 7.05 (d, 1H, CH), 3.02 (t, 2H, CH_2_), 2.69 (t, 2H, CH_2_), 2.39 (s, 3H, CH_3_), 1.92 (m, 4H, 2CH_2_); GC-MS *m/z*: 180 (M^+^); Calcd for C_10_H_12_OS: C, 66.63; H, 6.71; S, 17.79. Found: C, 66.57; H, 6.63; S, 17.71.

### 5.5. General procedure for the synthesis of Mannich bases **12a,b**

Acetic anhydride (39 mmol) was added dropwise to a solution of dimethylamine hydrochloride (10 mmol) and 37% formaldehyde (29 mmol) at 85-90 °C and the mixture was stirred for 0.5 h. Then tetrahydrocyclohepta[*b*]thiophen-4-one **11a,b** (7 mmol) was added to the mixture and the whole stirred at 75 °C for 3h. After cooling, the mixture was evaporated under reduced pressure and the resulting crude residue was crystallized from acetone (**12a**) or triturated with diisopropyl ether (**12b**) to afford the desired product.

*5,6,7,8-Tetrahydro-5-dimethylaminomethyl-4H-cyclohepta[b]thiophen-4-one-HCl* (**12a**): Yield 0.76 g (42%) as a crystalline solid; mp 155 °C; *R_f_*: 0.23 (CHCl_3_-MeOH 9:1); IR (film, ν, cm^-1^): 1650 (CO); ^1^H-NMR (CDCl_3_), *δ* ppm: 7.37 (d, 1H, *J =* 5.4 Hz, CH), 7.04 (d, 1H, *J =* 5.4 Hz, CH), 4.02-3.59 (m, 2H, CH_2_-N^+^H (CH_3_)_2_), 3.30-3.00 (m, 3H, CH-5, CH_2_-8), 2.77 (m, 6H, 2CH_3_). 2.40-1.52 (m, 4H, 2CH_2_); GC-MS *m/z*: 259 (M^+^); Calcd for C_12_H_18_ClN OS; C, 55.48; H, 6.98; Cl, 13.65; S, 12.34. Found: C, 55.57; H, 6.92; Cl, 13.69; S, 12.39.

*2-Methyl-5,6,7,8-tetrahydro-5-dimethylaminomethyl-4H-cyclohepta[b]thiophen-4one-HCl* (**12b**): Yield 0.14 g (51%) as a crystalline solid: mp 156 °C; *R_f_*: 0.72 (CHCl_3_-MeOH 9:1); IR (Nujol), ν, cm^-1^): 1654 (CO); ^1^H-NMR (CDCl_3_), *δ* ppm: 6.96 (s, 1H, CH), 3.05 (t, 2H, CH_2_), 2.64 (t, 2H, CH_2_), 2.32 (s, 3H, CH_3_), 2.02 (m, 4H, 2CH_2_); GC-MS *m/z*: ND (M^+^); Calcd for C_13_H_20_ClNOS: C, 57.02; H, 7.36; Cl, 12.95; N, 5.12; S, 11.71. Found: C, 57.10; H, 7.39; S, 11.65. 

### 5.6. General procedure for the synthesis of nitriles **13a,b**

To a solution of the Mannich base **12a,b** (4 mmol) in methanol (8 ml), an aqueous solution of NaCN (22 mmol, 10 ml) was dropwise added, at RT, and the mixture was stirred at 55 °C for 4 h, then poured onto cold H_2_O and afterwards extracted with CH_2_Cl_2_ (3 × 5 mL). The resulting organic layer was washed with H_2_O, brine, dried (Na_2_SO_4_), filtered and evaporated *in vacuo*. 

*5,6,7,8-Tetrahydro-5-cianomethyl-4H-cyclohepta[b]thiophen-4-one* (**13a**): Yield 0.62 g (75%) as a dark oil; *R_f_*: 0.86 (CHCl_3_-MeOH 9:1); IR (film, ν, cm^-1^): 1660 (CO), 2246 (CN); ^1^H-NMR (CDCl_3_), *δ* ppm: 7.43 (d, 1H, *J =* 5.4 Hz, CH), 7.03 (d, 1H, *J =* 4.8 Hz, CH), 3.38-2.80 (m, 4H, 2CH_2_), 2.7-2.5 (m, H, CH-5), 2.24-1.68 (m, 4H, 2CH_3_); GC-MS *m/z*: 205 (M^+^); Calcd for C_11_H_11_NOS: C, 64.36; H, 5.44; N, 6.82; S, 15.62. Found: C, 64.32; H, 5.49; N, 6.91; S, 11.69. 

*2-Methyl- 5,6,7,8-Tetrahydro-5-cianomethyl-4H-cyclohepta[b]thiophen-4-one* (**13b**): Yield 0.49 g (47%) as an amorphous dark solid: mp 77-78 °C; *R_f_*: 0.90 (CHCl_3_-MeOH 9:1); IR (Nujol, ν, cm^-1^): 1645 (CO), 2237 (CN); ^1^H-NMR (CDCl_3_), *δ* ppm: 7.01 (s, 1H, CH), 3.07 (t, 2H, CH_2_), 2.7 (m, 2H, CH_2_), 2.40 (s, 3H, CH_3_), 2.26 (m, 4H, 2CH_2_); GC-MS *m/z*: 219 (M^+^); Calcd for C_12_H_13_NOS: C, 65.72; H, 5.97; N, 6.39; S, 14.62. Found: C, 65.72; H, 5.93; S, 14.67. 

### 5.7. General procedure for the synthesis of acids **14a,b**

To a solution of nitrile **13a,b** (3.5 mmol) in AcOH (3.6 ml), HCl conc. (2.5 ml) was dropwise added at RT, then the reaction mixture was refluxed for 3 h (TLC). After cooling to RT, the mixture was diluted with cold H_2_O and afterwards extracted with CH_2_Cl_2_ (4 × 5 mL). The resulting organic layer was washed with H_2_O, brine, dried (Na_2_SO_4_), filtered and evaporated *in vacuo*.

*4-Oxo- 5,6,7,8-tetrahydro-4H-cyclohepta[b]thiophen-5-acetic acid* (**14a**): Yield 0.67 g (85%) as a brown dark solid: mp145-147 °C; *R_f_*: 0.53 (CHCl_3_-MeOH 9:1); IR (Nujol, ν, cm^-1^): 1660 (CO), 1707 (COOH); ^1^H-NMR (CDCl_3_), *δ* ppm: 8.27 (bs, 1H, COOH, exchanged with D_2_O), 7.33 (d, 1H, *J =* 5.2 Hz, CH), 6.92 (d, 1H, *J =* 5.4 Hz, CH), 3.36 (m, 5H, 2CH_2_, CH-5), 2.63-1.57 (m, 4H, 2CH_2_); GC-MS *m/z*: 224 (M^+^); Calcd for C_11_H_12_O_3_S: C, 58.91; H, 5.38; S, 14.30. Found: C, 58.97; H, 5.43; S, 14.38.

*2-Methyl-4-oxo-5,6,7,8-tetrahydro-4H-cyclohepta[b]thiophen-5-acetic acid* (**14b**): Yield 0.88 g (82%) as a coffee-black solid: mp 145-147 °C. *R_f_*: 0.68 (CHCl_3_-MeOH 9:1); IR (Nujol, ν, cm^-1^): 1660 (CO), 1708 (COOH); ^1^H-NMR (CDCl_3_), *δ* ppm: 8.27(bs, 1H, COOH, exchanged with D_2_O), 7.01 (s, 1H, CH), 3.07 (t, 2H, CH_2_), 2.7 (m, 2H, CH_2_), 2.40 (s, 3H, CH_3_), 2.26 (m, 4H, 2CH_2_); GC-MS *m/z*: 239 (M^+^); Calcd for C_12_H_14_O_3_S: C, 60.48; H, 5.92; S, 13.46. Found: C, 60.56; H, 5.98; S, 13.52. 

### 5.8. General procedure for the synthesis of pyridazinones **15a,b**

To the solution of acid **14a,b** (3 mmol) in anhydrous EtOH (10 mL), H_2_NNH_2_·H_2_O 80% (3 mmol) was added dropwise and the resulting mixture was refluxed for 3 h. After cooling at room temperature, the solvent was evaporated *in vacuo.*

*2,4,4a,5,6,7-Hexahydro-3H-thieno[2',3':6,7]cyclohepta[1,2-c]pyridazin-3-one* (**15a**): Yield 0.59 g (87%) as a brown dark solid: mp 147-148 °C; *R_f_*: 0.66 (CHCl_3_-MeOH 9:1); IR (KBr, ν, cm^-1^): 1674 (CO), 3173 (NH); ^1^H-NMR (CDCl_3_), *δ* ppm: 8.89 (s, 1H, NH, exchanged with D_2_O), 7.26 (d, 1H, *J =* 4.2 Hz, CH), 7.04 (d, 1H, *J =* 5.4 Hz, CH), 3.10-2.6 (m, 4H, 2CH_2_), 2.70-2.20 (m, 3H, CH_2_, CH), 2.43-2.213(m, 2H, CH_2_-6), 2.17-1.63 (m, 3H, CH, CH_2_); GC-MS *m/z*: 220 (M^+^); Calcd for C_11_H_12_N_2_OS: C, 59.97; H, 5.49; N, 12.72; S, 14.56. Found: C, 59.92; H, 5.43; N, 12.67; S, 14.48. 

*9-Methyl- 2,4,4a,5,6,7-hexahydro-3H-thieno[2',3':6,7]cyclohepta[1,2-c]pyridazin-3-one* (**15b**): Yield 0.78 g (85%) as a colorless solid scales: mp 179-182 °C; *R_f_*: 0.68 (CHCl_3_-MeOH 9:1); IR (KBr, ν, cm^-1^): 1680 (CO), 3175 (NH); ^1^H-NMR (CDCl_3_), *δ* ppm: 8.39 (s, 1H, NH, exchanged with D_2_O), 3.10-2.82 (m, 4H, 2CH_2_), 2.70-2.20 (m, 3H, CH_2_, CH), 2.40 (s, 3H, CH_3_), 1.80-2.05 (m, 2H, CH_2_); GC-MS *m/z*: 234 (M^+^); Calcd for C_12_H_14_N_2_OS: C, 61.51; H, 6.02; N, 11.96; S, 13.69. Found: C, 61.45; H, 6.07; N, 11.91; S, 13.63. 

### 5.9. General procedure for the synthesis of 2-N-substituted-2,4,4a,5,6,7-hexahydro-3H-thieno[2’,3’:6,7]cyclohepta[1,2-c]pyridazin-3-one derivatives **7a-l**

To a solution of pyridazinone (0.91 mmol) in anhydrous ethanol (5 mL), 37% formaldehyde (11 mmol) and appropriate amines (2 mmol) were added and the mixture was refluxed under nitrogen atmosphere for 8 h. The reaction mixture was cooled to RT, then the solvent was evaporated under reduced pressure and the residue was taken-up in water and extracted with chloroform (4 × 5 mL). The combined organic phase was dried (Na_2_SO_4_), filtered and concentrated *in vacuo*, to give a crude oil which was purified by flash chromatography (FC). 

*2-N-[(4-N-Phenylpiperazin-1-yl)methyl]-2,4,4a,5,6,7-hexahydro-3H-thieno[2’,3’:6,7]cyclohepta[1,2-c]pyridazin-3-one* (**7a**): FC: petroleum ether/EtOAc 6.5:3.5; Yield 0.19 g (52%) as a beige solid with mp 121-123 °C; *R_f_*: 0.35 (petroleum ether/EtOAc 6.5:3.5); IR: (KBr, ν, cm^-1^): 1661 (CO); ^1^H-NMR (CDCl_3_), *δ* ppm: 7.28 (d, 1H, *J =* 5.8 Hz, CH), 7.05 (d, 1H, *J =* 5.6 Hz, CH), 7.00-6.80 (m, 5H, Ar-H),4.84 (dd, 2H, *J =* 13.2 Hz, 2H, CH_2_), 3.38-3.12 (m, 4H, ArN(CH_2_)_2_,), 3.07-2.63 (m, 6H, , 2H, CH_2_-7,4H, N(CH_2_)_2_), 2.78-2.60 (m, 2H, CH_2_-4), 2.50-2.30 (m, 2H, CH_2_-6), 2.06-1.70 (m, 3H, CH-4a, CH_2_-5); GC-MS *m/z*: 395 (M^+^); Calcd for C_22_H_26_N_4_OS: C, 66.97; H, 6.64; N, 14.20; S, 8.13. Found: C, 66.85; H, 6.53; N, 13.99; S, 8.02.

*2-N-[(4-(o-Methoxy-phenylpiperazin-1-yl)methyl]-2,4,4a,5,6,7-hexahydro-3H-thieno[2’,3’:6,7]cyclo-hepta[1,2-c]pyridazin-3-one* (**7b**): FC: petroleum ether/EtOAc 6.5:3.5; Yield 0.186 g (48%) as a glassy solid with mp 50-51 °C; *R_f_*: 0.23 (petroleum ether/EtOAc 6.5:3.5); IR (Nujol, ν, cm^-1^): 1669 (CO); ^1^H-NMR (CDCl_3_), *δ* ppm: 7.20 (d, 1H, *J =* 5.4 Hz, CH-9), 6.94 (d, 1H, *J =* 5.4 Hz, CH-10), 6.90-6.70 (m, 4H, Ar-H), 4.75 (dd, 2H, CH_2_), 3.74 (s, 3H, OCH_3_), 3.15-2.75 (m, 10H, 5CH_2_, Ar-N(CH_2_)_2_, CH_2_, N(CH_2_)_2_; 2,70-2.20 (m, 4H, 2CH_2_), 2.00-1.60 (m, 3H, CH-4a, CH_2_-5); GC-MS *m/z*: 425 (M^+^); Anal. Calcd for C_23_H_28_N_4_O_2_S: C, 65.07; H, 6.65; N, 13.20; S, 7.55. Found: C, 64.86; H, 6.53; N, 13.09; S, 7.43.

*2-N-[(4-(o-Fluoro-phenylpiperazin-1-yl)methyl]-2,4,4a,5,6,7-hexahydro-3H-thieno[2’,3’:6,7]cyclo-hepta[1,2-c]pyridazin-3-one* (**7c**): FC: petroleum ether/EtOAc 6.5:3.5; Yield 0.217 g (58%) as a white solid with mp 170 °C; *R_f_*: 0.32 (petroleum ether/EtOAc 6.5:3.5); IR (Nujol, ν, cm^-1^): 1671 (CO); ^1^H-NMR (CDCl_3_), *δ* ppm: 7.28 (d, 1H, *J =* 5.6 Hz, CH), 7.04 (d, 1H, *J =* 5.6 H*z*, CH), 7.13-6.86 (m, 4H, Ar-H), 4.82 (dd, 2H, *J =* 13 Hz, CH_2_), 3.20-2.85 (m, 10H, ArN(CH_2_)_2_, CH_2_, N(CH_2_)_2_), 2.76-2.30 (m, 4H, 2CH_2_), 2.05-1.70 (m, 7H, 3H, CH_2_, CH); GC-MS*m/z*: 413 (M^+^); Calcd for C_22_H_25_FN_4_OS: C, 64.05; H, 6.11; F, 4.61; N, 13.58; S, 7.77. Found: C, 64.17; H, 6.03; N, 13.49; S, 7.65.

*2-N-[(4-(o-Methylpiperazin-1-yl)methy]-2,4,4a,5,6,7-hexahydro-3H-thieno[2’,3’:6,7]cyclohepta[1,2-c]pyridazin-3-one* (**7d**): FC: petroleum ether/EtOAc 6.5:3.5; Yield 0.020 g (5.3%) as a brown solid with mp 118 °C; *R_f_*: 0.32 (petroleum ether/EtOAc 6.5:3.5); IR (Nujol, ν, cm^-1^): 1673 (CO); ^1^H-NMR (CDCl_3_), *δ* ppm: 7.29 (d, 1H, *J =* 5.4 Hz, CH-9), 7.05 (d, 1H, *J =* 5.2 Hz, CH-10), 5.26 (m, 2H, CH_2_-a), 3.10-2.86 (m, 6H, CH_2_, N(CH_2_)_4_), 2.68-1.72 (m. 14H, N(CH_2_)_2_, 3CH_2_, CH_3_,CH); GC-MS *m/z*: 332 (M^+^); Calcd for C_17_H_24_N_4_OS: C, 61.41; H, 7.28; N, 16.85; S, 9.64. Found: C, 61.39; H, 7.13; N, 16.73; S, 9.48.

*9-Methyl-2-N-[(4-N-phenylpiperazin)methyl]-2,4,4a,5,6,7-hexahydro-3H-thieno[2’,3’:6,7]cyclo-hepta[1,2-c]pyridazin-3-one* (**7e**): FC: petroleum ether/acetone 6.5:3.5; Yield 0.070 g (20%) as a beige solid with mp 51-52 °C; *R_f_*: 0.42 (petroleum ether/EtOAc 6.5:3.5); IR (Nujol, ν, cm^-1^): 1671 (CO); ^1^H-NMR (CDCl_3_), *δ* ppm: 7.35-6.80 (m, 6H, 5H, Ar, 1H, CH-10), 4.81 (dd, 2H, *J =* 13,2 Hz, 2H, CH_2_-a), 3.35-3.08 (t, 4H, ArN(CH_2_)_2_,), 3.00-2.80 (m, 6H, 4H, N(CH_2_)_2_, 2H, CH_2_), 2.75-1.67 (m, 10H, 6H, CH_2_, 1H, CH_3_, 1H, CH); GC-MS *m/z*: 408 (M^+^); Calcd for C_23_H_28_N_4_OS: C, 67.61; H, 6.91; N, 13.71; S, 7.85. Found: C, 67.52; H, 6.56; N, 13.58; S, 7.79.

*9-Methyl-2-N-[(4-(o-methoxy-phenylpiperazin-1-yl)methyl]-2,4,4a,5,6,7-hexahydro-3H-thieno [2’,3’:6,7]cyclohepta[1,2-c]pyridazin-3-one* (**7f**): FC: petroleum ether/EtOAc 6.5:3.5; Yield 0.090 g (24%) as a glassy solid with mp 51-53 °C; R_f_: 0.23 (petroleum ether/EtOAc 6.5:3.5); IR (KBr, ν, cm^-1^): 1671 (CO); ^1^H-NMR (CDCl_3_), δ ppm: 6.97-6.65 (m, 5H, 4H, Ar, 1H, CH-10), 4.73 (dd, 2H, CH_2_), 3.74 (s, 3H, OCH_3_), 3.12-2.72 (m, 10H, 4H, ArN(CH_2_)_2_, 2H, CH_2_, 4H, N(CH_2_)_2_; 2,38 (s, 3H, CH_3_), 2.62-1.62 (m, 7H, 6H, CH_2_, 1H, CH); GC-MS m/z: 439 (M^+^); Calcd for C_24_H_30_N_4_O_2_S: C, 65.72; H, 6.89; N, 12.77; S, 7.31. Found: C, 65.79; H, 6.78; N, 12.65; S, 7.43.

*9-Methyl-2-N-[(4-(o-fluoro-phenylpiperazin-1-yl)methyl]-2,4,4a,5,6,7-hexahydro-3H-thieno [2’,3’:6,7]cyclohepta[1,2-c]pyridazin-3-one* (**7g**): FC: petroleum ether/EtOAc 6.5:3.5; Yield 0.217 g (47%) as a white solid with mp 138-140 °C; R_f_: 0.29 (petroleum ether/EtOAc 6.5:3.5); IR (KBr, ν, cm^-1^): 1665 (CO); ^1^H-NMR (CDCl_3_), δ ppm: 7.23-6.86 (m, 6H, 4H, Ar, 1H,CH-10), 4.81 (dd, 2H, CH_2_), 3.18-3.02 (t, 4H, ArN(CH_2_)_2_, 2.97-2.02 m, 6H, 4H, N(CH_2_)_2_, 2H, CH_2_), 2.41 (s, 3H, CH_3_),2.75-2.72 (m, 7H, 6H, CH_2_, 1H, CH); GC-MSm/z: 426 (M^+^); Calcd for C_23_H_27_F N_4_OS: C, 64.76; H, 6.38; F, 4.45; N, 13.13; S, 7.52. Found: C, 64.67; H, 6.25; F, 4.38; N, 13.19; S, 7.63.

*9-Methyl-2-N-[(4-methylpiperazin-1-yl)methy]-2,4,4a,5,6,7-hexahydro-3H-tieno*[2’,3’:6,7] *cyclohepta [1,2-c]pyridazin-3-one* (**7h**): FC: petroleum ether/EtOAc 6.5:3.5; Yield 0.012 g (5%) as a brown solid with mp 136- 137 °C; *R_f_*: 0.46 (ether); IR (Nujol, ν, cm^-1^): 1667 (CO); ^1^H-NMR (CDCl_3_), *δ* ppm: 6.97 (s, 1H, CH), 5.24 (dd, 2H, CH_2_), 3.15-2.84 (m, 6H, 3CH_2_), 2.41 (s, 3H, CH_3_), 2.28 (s, 3H, CH_3_), 2.73-2.22 (m, 6H, 3CH_2_), 2.10-1.72 (m, 5H, 2CH_2_, CH ); GC-MS *m/z*: 347 (M^+^); Anal. Calcd for C_18_H_26_N_4_OS: C, 62.40; H, 7.56; N, 16.17; S, 9.25. Found: C, 62.35; H, 7.48; N, 16.24; S, 9.36.

*2-N-[(4-Methylpiperidin-1-yl)methy]-2,4,4a,5,6,7-hexahydro-3H-thieno[2’,3’:6,7]cyclohepta[1,2-c]- pyridazin-3-one* (**7i**): FC: petroleum ether/EtOAc 6.5:3.5; Yield 0.17 g (45%) as a white solid with mp 87 °C; *R_f_*: 0.33 (petroleum ether/EtOAc 6.5:3.5); IR (Nujol, ν, cm^-1^): 1659 (CO); ^1^H-NMR (CDCl_3_), *δ* ppm: 7.2 (d, 1H, *J =* 5.0 Hz, CH-9), 7.3 (d, 1H, *J =* 5.2 Hz, CH-10), 4.74 (dd, 2H, *J =* 12.8 Hz, CH_2_), 3.13-2.25 (m, 8H, CH_2_-7, N(CH_2_)_2_, CH_2_-4), 2.05-1.18 (m, 10H, 4CH_2_, 2CH), 0.91 (d, 3H, CH_3_); GC-MS *m/z*: 331 (M^+^); Calcd for C_18_H_25_N_3_OS: C, 65.22; H, 7.60; N, 12.68; S, 9.67. Found: C, 65.15; H, 7.48; N, 12.57; S, 9.49.

*9-Methyl-2-N-[(4-methylpiperidin-1-yl)methy]-2,4,4a,5,6,7-hexahydro-3H-thieno[2’,3’:6,7]cyclohepta [1,2-c]pyridazin-3-one* (**7j**): FC: petroleum ether/ethyl acetate 6.5:3.5; Yield 0.190 g (51%) as a white solid with mp 133-135 °C; *R_f_*: 0.42 (petroleum ether/EtOAc 6:4); IR (Nujol, ν, cm^-1^): 1667 (CO); ^1^H-NMR (CDCl_3_), *δ* ppm: 6.92 (s, 1H, CH),4.73 (dd, 2H, *J = 15.8* Hz, CH_2_), 3.12-2.25 (m, 8H, 4CH_2_), 2.41 (s, 3H, CH_3_), 2.05-1.18 (m, 10H, 4CH_2_, 2CH), 0.91 (d, 3H, CH_3_); GC-MS *m/z*: 345 (M^+^); Calcd for C_19_H_27_N_3_OS: C, 66.05; H, 7.88; N, 12.16; S, 9.28. Found: C, 66.15; H, 7.78; N, 12.08; S, 9.36.

*2-N-[(4-Morpholine-1-yl)methyl]-2,4,4a,5,6,7-hexahydro-3H-thieno[2’,3’:6,7]cyclohepta[1,2-c] pyridazin-3-one* (**7k**): FC: petroleum ether/ acetone 6.5:3.5; Yield 0.23 g (78%) as a brown solid with mp 80 °C; *R_f_*: 0.44 (petroleum ether/acetone 7:3); IR (Nujol, ν, cm^-1^): 1666 (CO); ^1^H-NMR (CDCl_3_), *δ* ppm: 7.25 (d, 1H, *J =* 4.8 Hz, CH-9), 7.04 (d, 1H, *J =* 5Hz, CH-10), 4.73 (dd, 2H, *J =* 13.2Hz, CH_2_), 3.7 (t, 4H, *J =* 4.4 Hz, O(CH_2_)_2_), 3.10 (m, 11H, CH_2_-7, N(CH_2_)_2_, CH_2_-4, CH_2_-6, CH-4a), 2.00-1.75 (m, 2H, CH_2_-5); GC-MS *m/z*: 319 (M^+^); Calcd for C_16_H_21_N_3_O_2_S: C, 60.16; H, 6.63; N, 13.16; S, 10.04. Found: C, 60.02; H, 6.56; N, 13.02; S, 9.89.

*9-Methyl-2-[(4-morpholine-1-yl)methyl]-2,4,4a,5,6,7-hexahydro-3H-thieno*[2’,3’:6,7] *cyclohepta[1,2-c]pyridazin-3-one* (**7l**): FC: petroleum ether/acetone 6.5:3.5; Yield 0.180 g (46%) as a white solid with mp 108-110 °C; *R_f_*: 0.46 (petroleum ether/acetone 7:3); IR (KBr, ν, cm^-1^): 1671 (CO); ^1^H-NMR (CDCl_3_), *δ* ppm: 6.90 (s, 1H, CH), 4.72 (dd, 2H, *J =* 14.6 Hz, CH_2_), 3.69 (t, 4H, *J =* 4.4 Hz, 2CH_2_), 2.91 (t, 2H, *J =* 6.6 Hz, CH_2_), 2.71 (t, 4H, *J =* 4.2 Hz, 2CH_2_), 2.41 (s, 3H, CH_3_), 2.64- 1.78 (m, 7H, 3CH_2_ CH); GC-MS *m/z*: 333 (M^+^); Calcd for C_17_H_23_N_3_O_2_S: C, 61.23; H, 6.95; N, 12.60; S, 9.62. Found: C, 61.15; H, 6.84; N, 12.54; S, 9.51.

## References

[1] Fidler I.J. (2003). The pathogenesis of cancer metastasis: the ‘seed and soil’ hypothesis revisited. Nat. Rev. Cancer.

[2] Chabner B.A., Amrein P.C., Druker B.J., Michaelson M.D., Mitsiades C.S., Goss P.E., Ryan D.P., Ramachandra S., Richardson P.G., Supko J.G., Wilson W.H., Brunton L.L., Lazo J.S., Parker K.L. (2005). Antineoplastic Agents. Goodman & Gilman’s the Pharmacological Basis of Therapeutics.

[3] Gilchrest B.A., Eller M.S. (2009). Cancer therapeutics: Smart and smarter. Drugs Future.

[4] Tišler M., Stanovnik B., Katritzky A.R., Rees C.W. (1984). Pyridazines and their Benzo Derivatives. Comprehensive Heterocyclic Chemistry, The Structure, Reactions, Synthesis and Uses of Heterocyclic Compounds.

[5] Pinna G.A., Murineddu G., Murruzzu C., Zuco V., Zunino F., Cappelletti G., Artali R., Cignarella G., Solano L., Villa S. (2009). Synthesis, modelling, and antimitotic properties of tricyclic systems characterised by a 2-(5-Phenyl-1*H*-pyrrol-3-yl)-1,3,4-oxadiazole moiety. Chem. Med. Chem..

[6] Cignarella G., Barlocco D., Pinna G.A., Loriga M., Curzu M.M., Tofanetti O., Germini M., Cazzulani P., Cavalletti E. (1989). Synthesis and biological evaluation of substituted benzo[*h*]cinnolinones and 3*H*-benzo[6,7]cyclohepta[1,2-*c*]pyridazinones: higher homologues of the antihypertensive and antithrombotic 5*H*-indeno[1,2-*c*]pyridazinones. J. Med. Chem..

[7] Cignarella G., Barlocco D., Curzu M.M., Pinna G.A., Cazzulani P., Cassin M., Lumachi B. (1990). Synthesis and pharmacological evaluation of 4,4a-dihydro-5*H*-[1]benzopyrano[4,3-*c*]pyridazin-3(2*H*)-ones bioisosters of antihypertensive and antithrombotic benzo[*h*]cinnolinones. Eur. J. Med. Chem..

[8] Pinna G.A., Curzu M.M., Fraghì P., Gavini E. (1996). Synthesis and pharmacological evaluation of 5,6-dihydrobenzo[*f*]cinnolin-2(3*H*)ones analogues of antihypertensive and antiaggregating benzo[*h*]cinnolinones. Farmaco.

[9] Pinna G.A., Salis E., Berta D., Gavini E. (1997). Synthesis and pharmacological evaluation of 4a-methyl-4,4a,5,6-tetrahydrothieno[2,3-*h*]cinnolin-3(2*H*)-ones. Farmaco.

[10] Tanaka H., Kirihara S., Yasumatsu H., Yakushiji T., Nakao T. (1997). Synthesis and evaluation of novel 2-aryl-2,5,6,7-tetrahydro-3*H*-thieno [2′,3′:6,7]cyclohepta[1,2-*c*]pyridazin-3-ones and 2-aryl-5, 6-dihydrothieno[2,3-*h*]cinnolin-3(2*H*)-ones as anxiolytics. Eur. J. Med. Chem..

[11] Pinna G.A., Curzu M.M., Murineddu G., Chelucci G., Cignarella G., Menta E., Krell H.W., Rastelli G., Ferrari A.M. (2000). Preparation of thieno[3,2-*h*]cinnolinones as matrix metalloproteinase inhibitors. Arch. Pharm. Pharm. Med. Chem..

[12] Costantino L., Rastelli G., Vescovini K., Cignarella G., Vianello P., Corso A.D., Cappiello M., Mura U., Barlocco D. (1996). Synthesis, activity, and molecular modeling of a new series of tricyclic pyridazinones as selective aldose reductase inhibitors. J. Med. Chem..

[13] Pau A., Asproni B., Boatto G., Grella G.E., De Caprariis P., Costantino L., Pinna G.A. (2004). Synthesis and aldose reductase inhibitory activities of novel thienocinnolinone derivatives. Eur. J. Pharm. Sci..

[14] Pirisi M.A., Murineddu G., Mussinu J.M., Pinna G.A. (2002). Synthesis and cytotoxicity evaluation of thiophene analogues of 1-methyl-2, 3-bis(hydroxymethyl)benzo[g]indole bis[N-(2-propyl)carbamate]. Farmaco.

[15] Grella G.E., Cabras M.C., Murineddu G., Pau A., Pinna G.A. (2003). Synthesis and cytotoxicity of substituted 2-benzylnaphth[2,3-d]imidazoles. Eur. J. Pharm. Sci..

[16] Murineddu G., Cignarella G., Chelucci G., Loriga G., Pinna G.A. (2002). Synthesis and cytotoxic activities of pyrrole[2,3-d]pyridazin-4-one derivatives. Chem. Pharm. Bull..

[17] Silverman R.J. (2004). The Organic Chemistry of Drug Design and Drug Action.

[18] Monks A., Scudiero D., Skehan P., Shoemaker R., Paull K.D., Vistica D., Hose C., Langley J., Cronise P., Vaigro-Wolff A. (1991). Feasibility of a high-flux anticancer drug screen using a diverse panel of cultured human tumor cell lines. J. Natl. Cancer Inst..

[19] Paull K.D., Shoemaker R.H., Hodes L., Monks A., Scudiero D.A., Rubinstein L., Plowman J., Boyd M.R. (1989). Display and analysis of patterns of differential activity of drugs against human tumor cell lines: development of mean graph and COMPARE algorithm. J. Natl. Cancer Inst..

[20] Boyd M.R., Paull K.D. (1995). Some practical considerations and applications of the national cancer institute in vitro anticancer drug discovery screen. Drug Dev. Res..

[21] Boyd M.R., Teicher B.A. (1997). In Cancer Drug Discovery and Development.

[22] Block H.J., Beale J.M. (2004). Wilson and Gisvold’s Textbook of Organic Medicinal and Pharmaceutical chemistry.

